# “How to Select a Representative Product Set From Market Inventory?” A Multicriteria Approach as a Base for Future Reformulation of Cookies

**DOI:** 10.3389/fnut.2021.749596

**Published:** 2022-01-24

**Authors:** Carole Liechti, Julien Delarue, Isabelle Souchon, Véronique Bosc, Anne Saint-Eve

**Affiliations:** ^1^University of Paris Saclay, UMR SayFood, AgroParisTech, INRAE, Palaiseau, France; ^2^Department of Food Science and Technology, University of Davis, Davis, CA, United States; ^3^Avignon University, UMR SQPOV, INRAE, Avignon, France

**Keywords:** market inventory, multiple criteria, sample selection, food reformulation, nutrition, health

## Abstract

Consuming too much fat, sugar, and salt is associated with adverse health outcomes. Food reformulation is one possible strategy to enhance the food environment by improving the nutritional quality of commercial products. However, food reformulation faces many hindrances. One way to alleviate some of these hindrances is to embrace a multicriteria approach that is based on a market inventory. In this objective, additional sensory screening and water content analyses allow going beyond nutrition and composition information on the packaging. However, due to feasibility reasons for later in-depth analyses, it is necessary to work with several reduced and manageable products. To the best of the authors' knowledge, in the literature, there is no sample selection approach taking into account multiple criteria as a base for future food reformulation. The overall aim of this paper is to propose a method to select the best representative products from the market base, for future reformulation by going beyond nutrition and composition information on the packaging. This approach considered therefore nutrition, composition, economic, water content, and sensory information with the example of the cookies market. The first step is the creation of an extensive cookie database including sensory and water content information. In total 178 cookies among the French market were identified, then focus was placed on 62 chocolate chip cookies only. Sensory screening and water content analyses of all 62 products were conducted. The second step is to make an informed subset selection, thanks to a cluster analysis based on 11 nutrition, composition, and water content variables. A representative subset of 18 cookies could be derived from the obtained clusters. The representativity was evaluated with statistical uni- and multivariate analyses. Results showed a broad variety of chocolate chips cookies with a large nutritional, compositional, water content, and sensory differences. These results highlighted the first paths for future reformulation in this product category and showed the importance to include physical product information beyond the information on the packaging. This complete database on the selected cookies constituted a solid base for identifying future reformulation levers, in order to improve the nutritional quality and health.

## Introduction

Food reformulation is an interesting lever to reduce over-consumed nutrients (such as sugar, fat, and salt) and to enhance our diet and health (1, 2). A successful reformulation makes it possible to move toward a healthier food offer without largely changing consumers eating habits. It was shown that a reduction of undesirable nutrients such as trans fatty acids, saturated fat, sodium, and sugar of more than 17,000 foods and beverages would lead to a reduced intake of nutrients which are to limit and improve public health (3).

However, a recent study across 20 European countries showed that many packaged foods and drinks still have too high fat, sugar, and salt, as well as too low fiber content (4). These results of concern are surprising as the link between overconsumed adverse nutrients and negative health outcomes is well known (5). For example in the UK, most of the companies between 2015 and 2018 did not reach an overall sugar reduction of 5% among the top five product categories which contribute the most to the high sugar intake (biscuits, cereal bars, breakfast cereal, chocolate, and sugar confectionery, yogurts) among the UK population (6).

These findings underline the fact that food reformulation still has a lot of unused potentials, very likely due to many technological and sensory barriers. Indeed, reformulating biscuits is challenging. For instance, decreasing sugar and fat content is difficult because of their multiple functional properties in the food matrix, and in particular in sweet bakery products (7–9). Moreover, sugar and fat are strong drivers of preferences. Any modification might have huge consequences on liking, pleasure experiences, and food choices (10, 11). This may contribute to the hesitant willingness for voluntary food reformulation on the part of the food industry as cost and time effectiveness are not immediately granted.

To overcome these barriers and to encourage industries to reformulate healthier versions of their products, we argue that is necessary to see food reformulation in a comprehensive way because of the multifactorial nature of the determinants of food preferences and the multiple interactions between food components in the food matrix (10–14). Focusing only on nutritional and compositional changes would thus inevitably lead to dead-ends or missed opportunities. Improving the nutrition quality by food reformulation is complex and needs to integrate different dimensions such as food composition, physicochemical properties, and sensory perception. To address this challenge, it would be useful to create a solid base relying on multiple criteria in order to anticipate possible interactions and to achieve a successful reformulation in the long term.

Biscuits structure is highly dependent on processing conditions (temperature, moisture, time) and formulation (presence of sugars and fats) (15). Water content is thus a key property for biscuits' structure, texture, and fracture properties. For example, biscuits with a lower water content tend to show a higher measured fracture than biscuits with higher water content. In other words, biscuits with a higher water content tend to be softer than biscuits with lower water content (16, 17). As a result water content may indirectly drive biscuit preferences (15). In addition, water content is very important for products stability and shelf-life (18).

Besides the above-mentioned relationship between cookies' water content, structure, and texture, it is further very important to better understand consumers' perception of cookies' texture.

A sensory analysis (temporal dominance of sensation) with a trained panel showed that “hardness” was the first dominant attribute resulting from biscuits formulations (19). Interestingly, fat and/or sugar reduction leads to an increased hardness of the biscuits (20, 21). Therefore, we assume that the sensory variable “hardness” is an important attribute when it comes to food reformulation among a complex (high sugar and fat at the same time) food matrix, such as sweet bakery products.

Moreover, biscuits can usually be divided into two subcategories: soft or crunchy. In some cases, this information is available on the front packaging. However, some brands could have an intermediate texture, therefore a texture somewhere between hard and soft. Due to his missing texture information, it is thus impossible for the consumers to know in advance what type of cookie texture they buy until they actually eat them (or at least open the package and manipulate the cookies).

This article proposes a guide on how to create such a base for future reformulation. Cookies were chosen as a case study of prime relevance. The first aim was to create a database with the help of comprehensive market inventory, taking into account multiple criteria by going beyond nutrition and composition information on the packaging. This multicriteria approach considers easily available information from the packaging (nutrition, composition, and price) and adds complementary information obtained thanks to simple analyses such as water content measurement and sensory screening.

However, conducting any advanced measurement with such a high number of products would be very time-intensive. For example, developing a protocol for any physicochemical analysis for all the products with their large product diversity is complex, especially when conducting the measurements in triplicates. The same goes for sensory panelists. Testing such a high number of products would be too demanding and might be physically impossible, because of sensory fatigue, or increased number of evaluation sessions.

This approach aims to provide a holistic view of the market which could benefit the industries by evaluating their competitors' products and ultimately gaining a better understanding of their own product positioning. But the food market is complex to analyze, with many different recipes from many different manufacturers. An investigation of the product category under consideration is thus a first necessary step to identify the diversity of existing products. A sensory screening and a water content analysis of all included cookies were thus necessary to get the first-level view of product texture. Then, further in-depth analyses are usually needed to gain greater knowledge of the existing recipes and to guide reformulation. To make these analyses realistic and compatible with experimental constraints, a second step in the proposed approach is to define a subset of products that would be representative of the market and yet be of manageable size. In this objective, we suggest making an informed selection based on a multicriteria analysis. To the best of the authors' knowledge, there is a lack of a common subset selection method adapted to food reformulation and that takes into account multiple variables.

## Materials and Methods

**Figure 1:**
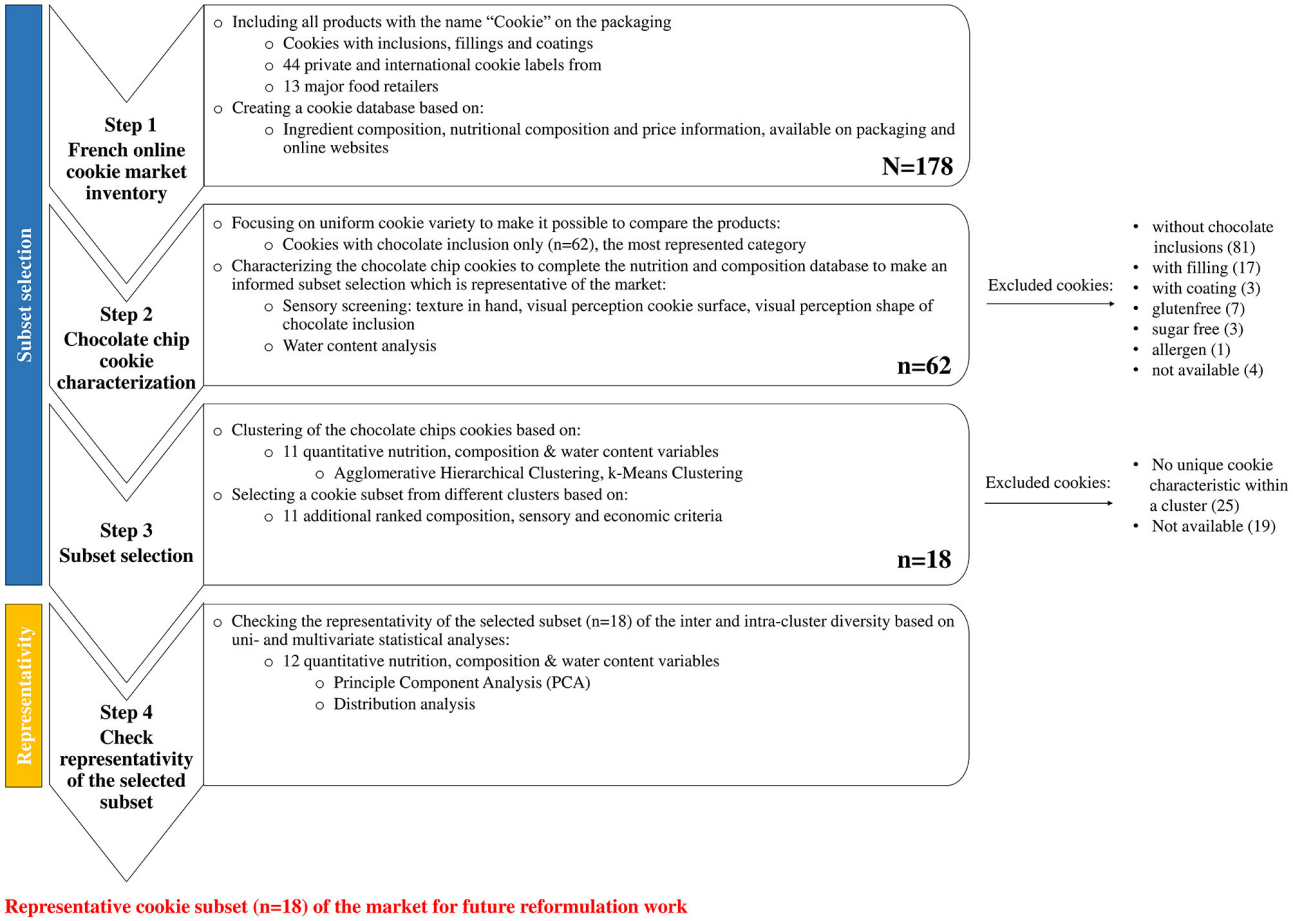
Overview of the four steps from the online cookie market inventory to the selected subset and its check of the representativity.

This section describes the four steps used for this multicriteria approach, from the analysis of the cookie market to the subset selection and the representativity checks (Figure 1, steps 1–4). First of all, to identify the potential for reformulation and to explore the diversity of recipes of the “commercial cookies” product category, online analysis of the French market was conducted (step 1). The focus was set on a uniform cookie variety. In order to identify possible levers for food reformulation in a later step, it is important to first have a broad view of products' characteristics. For this, we needed to select a representative subset of products while maintaining a good vision of the market diversity. Therefore, sensory screening and water content analyses were first conducted on all chocolate chip cookies (step 2). Then, the cookies were clustered based on available nutrition, composition, and water content data. A subset of products was then proposed based on additional 11 compositions, and sensory and economic criteria ranked for their importance in the selection (step 3). Finally, a check of the subset representativity was performed with uni- and multi-dimensional statistical analyses (step 4).

### Cookie Market Inventory (Step 1)

All the cookie brands available in 2019 on the online French market with the specific mention “Cookie” on the packaging were included in this study (Figure 1, step 1). All the different varieties were initially considered, including cookies with inclusions, fillings, and coatings and specialites such as gluten allergen and sugar free products. A database with a total of 178 cookies was created, using information from the packaging and websites. Available nutrition, composition, and price information from the packaging and online websites were collected. In addition to that, the Nutri-Score (letters A-E) was calculated based on the Rayner computation for each product (22).

#### Focus on Uniform Cookie Category: Chocolate Chip Cookies

We aimed to focus on a uniform cookie variety in order to work on a homogeneous product set and to make future reformulation work consistent. Cookies with chocolate inclusions showed the broadest range and greatest diversity within the market offer. Therefore, the decision was made to consider cookies containing chocolate inclusions only. Besides, it is worth noting that chocolate chips are important carriers of sugar and fat which makes them a potentially interesting lever for reformulation.

We excluded all cookies without chocolate inclusions, those with fillings and coatings, and specialties cookies such as gluten-free, sugar-free, and allergen-free cookies. Finally, a total of 62 cookies with chocolate inclusions were included in this study (Figure 1, step 2 excluded cookies).

#### Variables and Criteria Among the Chocolate Chip Cookie Database

Besides the cookie information from the packaging and online websites, the chocolate chip cookie database was further completed with water content analysis (section Water Content) and sensory screenings (visual perception shape chocolate inclusion, and cookie surface) (sections Screening of the Texture in Hand – Screening of the Cookie Surface and the Shape of the Chocolate Inclusion) of the 62 cookies. All quantitative variables were included for the cluster analyses [section Subset Selection (Step 3) and Cookie Clustering (Step 3)] and representativity checks [sections Statistical Representativity Check (Step 4) and Validation With Multi-Dimensional Statistical Analysis (Step 4)], whereas additional ranked criteria were considered for the subset selection among clusters (section Additional Ranking Criteria). All variables and criteria used for the chocolate chip cookies database are presented in Supplementary Tables 1, 2.

##### Quantitative Variables

In this method, 14 quantitative nutrition, composition, and water content variables were used to constitute the database: kcal, fat, saturated fat, carbohydrates, sugar, protein, fiber and salt content per 100 g, number of technological additives (important for baking and conservation properties such as baking powder, emulsifier, thickening agents, antioxidants, and humectant), number sensory additives (important for sensory properties such as colorings and artificial sweeteners), number additives (technological and sensory additives), number ingredients, water content, and the calculated Rayner score.

Furthermore, 11 variables (fat, saturated fat, carbohydrates, sugar, protein, fiber, and salt content per 100 g, number technological additives, number sensory additives, number ingredients, and water content) were included for the clustering [section Subset Selection (Step 3)], while 12 variables [kcal, fat, saturated fat, carbohydrates, sugar, protein, fiber and salt content per 100 g, number additives (technological and sensory additives), number ingredients, water content, and the calculated Rayner score] were included for the representativity check [section Statistical Representativity Check (Step 4)].

##### Additional Ranking Criteria

**Figure 2:**
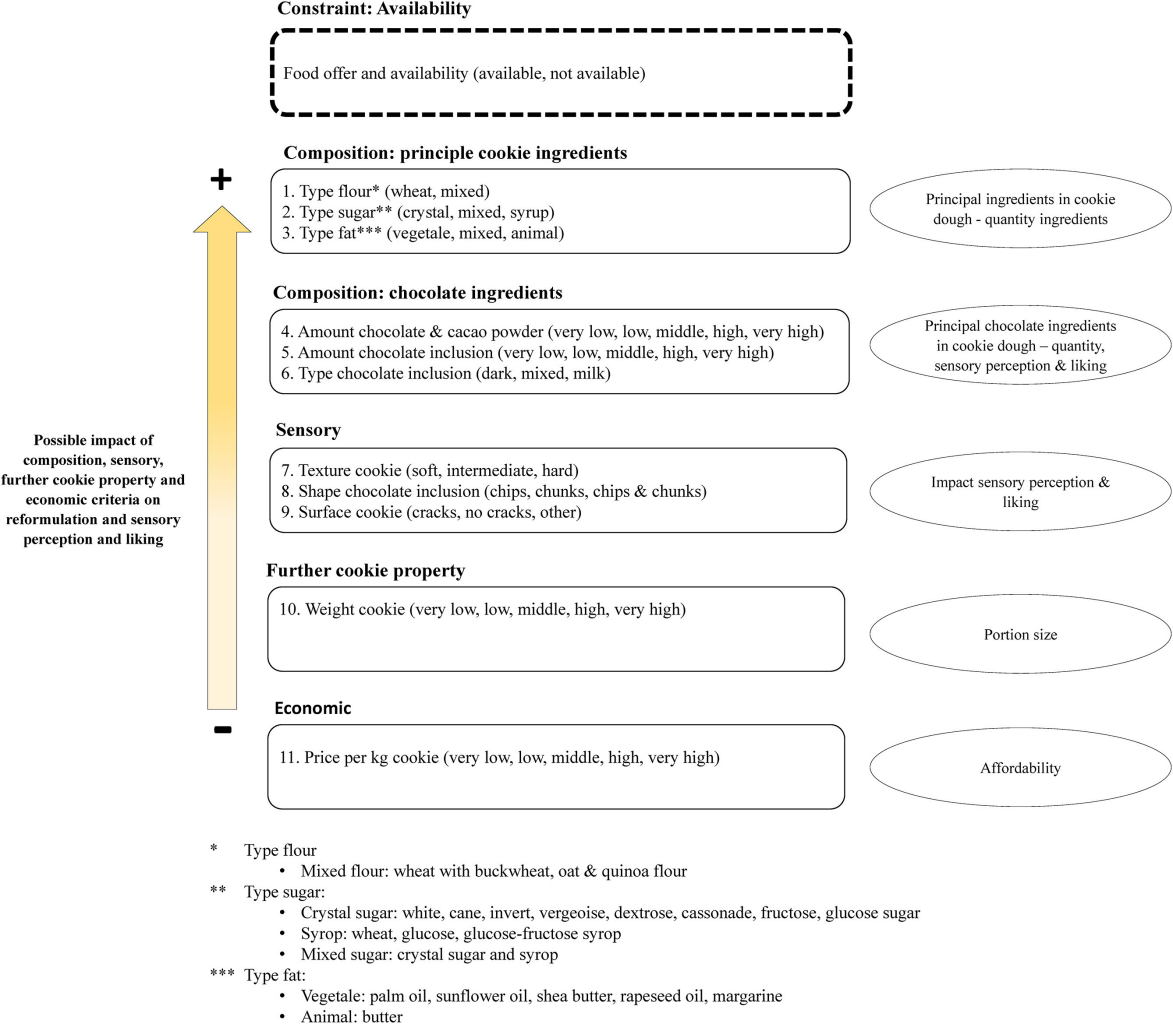
Overview of the 11 additional ranked criteria with their 40 subgroups (in brackets) and the availability as constraint.

Additional ranked compositions, sensory and economic criteria with their 40 subgroups were also included in the database (Figure 2). Cookies' availability was not included as a criterion but as a constraint, as for obvious practical reasons products must be available for further analyses.

All 11 criteria were ranked according to their possible impact on the food structure, sensory perception, and liking. In addition to these criteria, product availability at the time of the study was a strong constraint and was taken into account for the final selection.

Obviously, composition criteria such as major cookies ingredients and chocolate ingredients were considered as most impactful on the food matrix due to their high quantity in the recipe.

Cookies' weight and price were included as well. These two criteria lesser impact reformulation, sensory perception, and liking. However, the cookie weight might play an important role in portion size and kcal intake. Besides, as healthier reformulated products should be available for all, we also included the price that is an important determinant of purchasing behavior.

Moreover, six criteria contained qualitative information (type of flour, type of sugar, type of fat, type of chocolate inclusion, shape of chocolate inclusion, and surface cookie), whereas five criteria contained quantitative information (amount of chocolate and cacao powder, amount of chocolate inclusion, texture cookie, weight of cookie and price per kg cookie).

All 11 qualitative and quantitative criteria were considered as categorial criteria with subgroups. For the 5 quantitative criteria, we calculated their quintile rank in order to obtain five subgroups of equal frequencies such as very low, low, middle, high, and very high. For the criterion texture in hand, we calculated the tertile rank for the score in order to distinguish between three main textures (soft, intermediate, and hard) (Table 1).

**Table 1:** Rank calculation of the quintiles and tertile of the subgroups.

**Criteria (quintiles)**	**Very low (%)**	**Low (%)**	**Middle (%)**	**High (%)**	**Very high (%)**
Amount chocolate and cocoa powder in % (per 100 g)	1.3–2.3	2.4–2.6	2.7–3.1	3.2–5.7	5.8–15.3
Amount chocolate inclusion in % (per 100 g)	5.5–22.6	22.7–28	28.1–30.5	30.6–37	37.1–40
**Criteria (quintiles)**	**Very low (g)**	**Low (g)**	**Middle (g)**	**High (g)**	**Very high (g)**
Weight cookie in g	3.12–16.5	16.6–16.6	16.7–23	23.1–25	25.1–70
**Criteria (quintiles)**	**Very low (€)**	**Low (€)**	**Middle (€)**	**High (€)**	**Very high (€)**
Price per kg cookie	2.9–4.5	4.6–7.5	7.6–10.4	10.5–16.7	16.8–29.3
**Criterion (tertile)**	**Soft**	**Intermediate**	**Hard**		
Texture in hand (score 0–10)	0–5	5.1–7.2	7.3–9.4		

### Chocolate Chip Cookie Characterization (Step 2): Sensory Screening and Water Content Analysis

Further analyses were conducted in order to go beyond composition and nutritional values from the packaging and to better understand the major sensory and water content characteristics of the products (Figure 1, step 2). Three sensory screenings (perceived texture in hand; visual perception cookie surface and shape of chocolate inclusion) were performed on all 62 chocolate chip cookies. The goal of the sensory screening was to categorize the cookies according to their most striking visual and texture characteristics. This type of evaluation is relatively easy and differences between the cookies were expected to be quite obvious. Under such conditions, it is thus possible to reach sufficient power, even with a very small number of panelists. Besides the sensory screening, the water content was measured for all 62 chocolate chip cookies. Measuring the water content among baked bakery products will provide information about cookies structure and texture characteristics.

#### Screening of the Texture in Hand

In order to gain insights into product texture, three trained subjects evaluated the hardness of all chocolate chip cookies. They were told to break the cookies in two halves by hand and to report their perceived hardness on an unstructured scale from 0 to 10, where 0 indicated soft and 10 hard. The evaluation took place over six sessions and was conducted in sensory booths in a sequential monadic way, following a balanced order over the panel and sessions. Samples were coded with random three-digit numbers and presented in blind.

#### Screening of the Cookie Surface and the Shape of the Chocolate Inclusion

Cookies' surface aspect and the shape of the chocolate inclusions were evaluated by a cookie expert who was trained over 1 year with all 62 chocolate chip cookies. The visually perceived cookie surface was grouped into the qualitative subgroups “cracks,” “no cracks,” and “other” which means neither cracks nor no cracks. The visually perceived shape of the chocolate inclusion was grouped into three qualitative subgroups “chips,” “chunks,” and “chips and chunks.”

#### Water Content

The water content was determined by the oven drying method and adapted from (23). First, all chocolate chip cookies were crushed and grinded with a mortar for 15 s. After grinding, 3 g of ground cookies were weighed in a round aluminum dish. It was further put in the oven (EM10, Chopin, France) for 18 h at 103°C. The time was set by 18 h as the weight after drying did not change anymore. The sample was then placed in a desiccator for 1 h before weighing. All measurements were performed in triplicates among three different cookies and the results averaged.

The mass loss was determined by weighing the sample before and after drying to constant weight:


$$\text{water}\;\text{content}\;\text{in}\;\%=\frac{\text{weight}\;(g)\;\text{cookie}\;\text{before}\;\text{oven}\;-\;\text{weight}\;(g)\;\text{cookie}\;\text{after}\;\text{oven}\;}{\text{weight}\;(\text{g})\;\text{cookie}\;\text{before}\;\text{oven}\;}*100$$

### Subset Selection (Step 3)

In order to best represent the market diversity and to make an informed subset selection, cookies were clustered based on 11 nutrition, composition, and water content variables defined in section Quantitative Variables (Figure 1, step 3). This allowed the selection of cookies from each cluster with different cookie characteristics.

In sensory science, Agglomerative Hierarchical Clustering (AHC) and K-means clustering are possible methods to group product characteristics or consumers in the same clusters based on their similarities and is, therefore, a suitable tool to contribute to decisions for product development (24, 25).

The subset selection from each cluster was done with the help of 11 additional ranked criteria defined in section Additional Ranking Criteria. As well other authors have used several ranked criteria for product selection (26, 27).

Within a cluster, those cookies with unique characteristics (subgroup) were selected. Please find more detailed information in Supplementary Table 3. Cookies that were marked as “not available” were excluded. The cookie numbers per cluster are shown in Supplementary Table 4.

### Statistical Representativity Check (Step 4)

In order to evaluate the representativity of the selected subset, we applied multi-dimensional analyses with 12 quantitative nutrition, composition, and water content variables (section Quantitative Variables). To check the subset based on their intra- and inter-cluster diversity, a principal component analysis (PCA) was performed on the 12 variables. To validate the representativity of the subset, we compared the distribution of the 62 chocolate chip cookies and the 18 selected cookies based on the 12 variables.

## Data Analysis

All statistical analyses (AHC, K-means ANOVA clustering, PCA, linear regression Histogram, and Linear Regression) were conducted with XLSTAT version 2018.1.1 (Addinsoft, New York, USA), where alpha = 0.05 was considered as the needed significance level.

### Cookie Clustering (Step 3)

To characterize and cluster cookies according to their nutrition, composition, and water content characteristics, we first ran an AHC with Euclidean distance, Wards' Method, and centered and reduced data. This analysis allows to visually define the optimal numbers of clusters. Data from the AHC were organized in a table with 11 columns (nutrition, composition, and water content analyses) and 62 rows (commercial chocolate chips).

Defining the “good” number of cookies clusters is a matter of balance between precision (the higher the number of clusters or products to be selected, the higher the precision of the selection) and feasibility (the higher the number of products, the more difficult to run additional analyses).

Once the number of clusters was selected, K-means clustering was applied (Trace (W) criterion on reduced and centered data the differences among the seven clusters was evaluated with an ANOVA). Conducting first an AHC followed by K-means is a common method in sensory science to obtain robust clusters.

### Validation With Multi-Dimensional Statistical Analysis (Step 4)

To visualize the seven clusters from the K-means clustering, the 10 active and two supplementary quantitative variables were plotted on a 2-dimensional map by using PCA. This allowed us to evaluate and check the multivariate nutrition, composition, and water content variables based on their intra- and inter-cluster diversity. For the PCA, we used Pearson correlation with a significance level alpha = 0.05 and standardized (n) data. Missing data were replaced by mean or mode. For the validation axes, F1 and F2 were considered. To evaluate the relationship between the texture in hand and cookies' water content, a linear regression was applied. The PCA is a convenient tool to visualize and plot obtained clustering data to detect class diversity (28).

To check the representativity of the selected subset, we visually compared the distributions of all 62 chocolate chip cookies and the 18 selected cookies based on the 12 quantitative variables thanks to histogram plots.

## Results

### Cookie Varieties and Compositional Diversity Among the Entire Cookie Database With Private and International Labels

**Figure 3:**
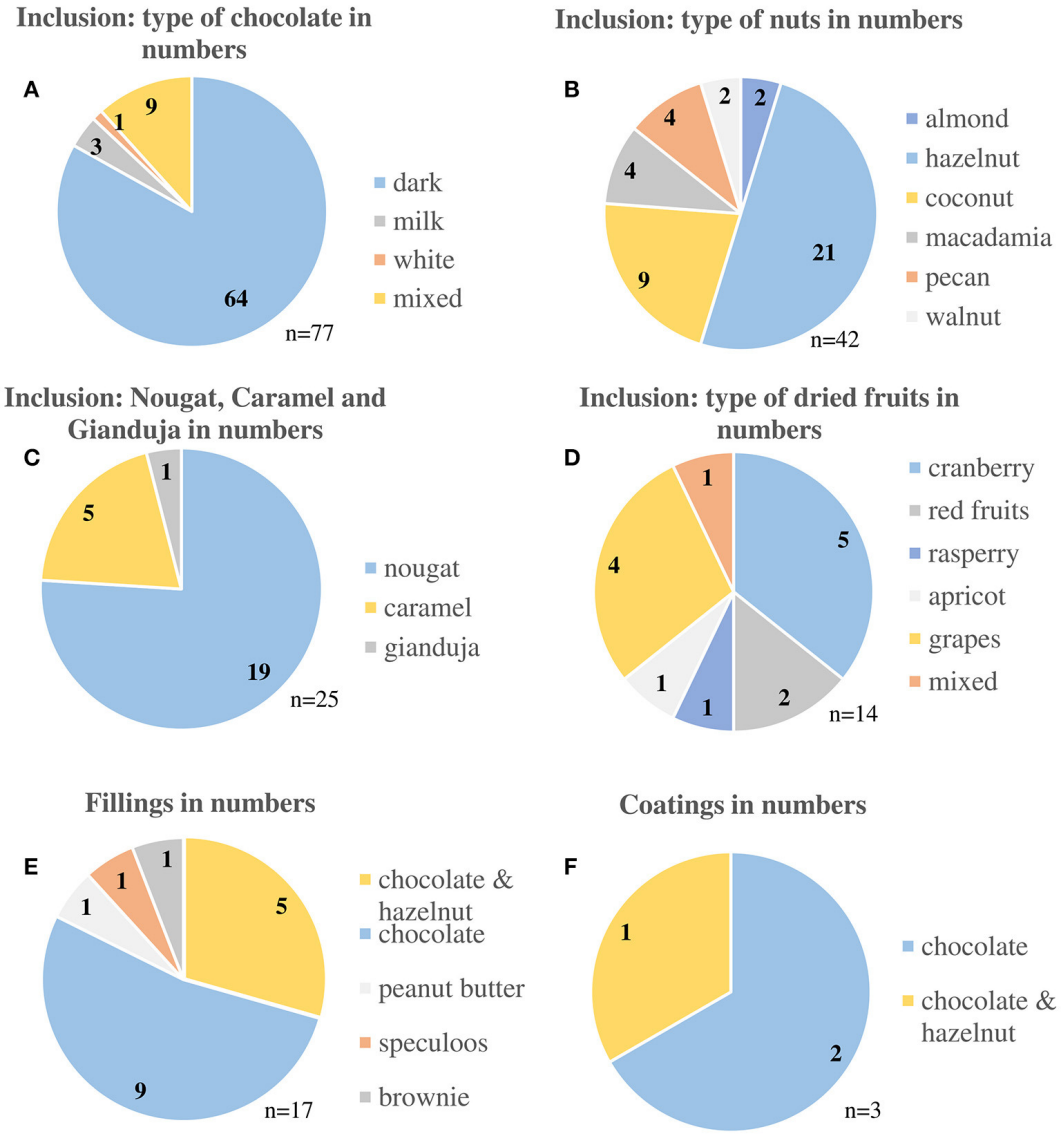
Cookie varieties from the online French market inventory in 2019. Cookies **(A–D)** are cookies with different inclusions and cookies **(E)** and **(F)** are cookies with fillings and coatings.

The results showed that three main cookie varieties were identified from the whole database with 178 cookies: 158 (88.8%) cookies with inclusions, 17 (9.5%) cookies with fillings, and 3 (1.7%) cookies with coatings. Among the cookies with inclusions, products with chocolate chips 77 (48.7%), nuts 42 (26.6%), nougat, caramel, and gianduja 25 (15.8%), and dried fruits 14 (8.9%) were the most represented cookies on the market. Around 20 (11.2%) cookies do have fillings and coatings, while more cookies have fillings than coatings. The vast majority of cookies on the French market thus have chocolate inclusions with dark chocolate or nuts inclusions with almonds. Figure 3 presents detailed information about the different inclusions, fillings, and coatings of commercial French cookies.

As shown in Table 2(A), the ranges (from the minimum to maximum values among the 178 cookies) for nutritional values (per 100 g) were: 183 kcal, 27 g sugar, 22.8 g carbohydrates, 16.4 g fat, and 16.4 g saturated fat. The calculated Rayner score showed that 170 (95.5%) cookies had a Nutri-Score of E, while 6 (3.3%) cookies had a Nutri-Score of D and 2 (1.2%) cookies had a Nutri-Score of B.

**Table 2:** Nutritional values of the entire- (A) and chocolate chip cookie database (B).

	**Databases A: 178 cookies B: 62 cookies**	**Min. value**	**Max. value**	**Difference Min. and Max. value**	**Mean ±SD**
Kcal	A	389	572	183	499.5 ± 25.5
	B	433	518	85	495.4 ± 15.2
Fat (g)	A	16	32.4	16.4	25.4 ± 3.1
	B	17.1	28	10.9	24.3 ± 2.0
Saturated fat (g)	A	3.6	20	16.4	12 ± 3.3
	B	5.9	18	12.1	12.6 ± 2.7
Carbohydrates (g)	A	48	70.8	22.8	59.6 ± 3.8
	B	57	70.8	13.8	61.6 ± 2.5
Sugar (g)	A	0*	43	27	32.1 ± 6.0
	B	27	41.8	14.8	34.5 ± 3.0
Protein (g)	A	3.5	13	9.5	6.2 ± 1.1
	B	4.5	7.6	3.1	6 ± 0.8
Fiber (g)	A	0.8	10	9.2	3.9 ± 1.5
	B	1.8	5.7	3.9	3.6 ± 0.9
Salt (g)	A	0.03	2	1.97	0.7 ± 0.4
* for sugar free cookies	B	0.2	1.5	1.3	0.8 ±0.3

* *0g of sugar per 100g was for the special cookies “sugar free” with artificial sweeteners*.

### Nutrition, Water Content, and Sensory Diversity Among the Chocolate Chip Cookie Database

In this study, we set the focus on 62 cookies with chocolate inclusions, representing 27 different private labels and international brands gathered from 12 retailers. As shown in Table 2(B), the broadest ranges for nutritional values per 100 g were found for kcal (85), sugar (14.8 g), carbs (13.8 g), saturated fat (12.1 g), and fat (10.9 g). Additionally, cookies with chocolate inclusion (B) demonstrated a slightly higher mean content of saturated fat, carbohydrates, sugar, and salt content, whereas we found a slightly lower mean content of protein and fiber compared to the whole cookie database (A). For cookies with chocolate inclusions, 61 cookies (98.4%) have a Nutri-Score E and only one (1.6%) cookie a Nutri-Score D. The Rayner score ranged between 14 (Nutri-Score D) and 28 (Nutri-Score E).

The mean value of the measured water content among the 62 cookies was 3.9 ± 1.7%, whereas the values ranged from min. 2.1% to max. 9.3%. For the perceived texture in hand, the mean value was 5.6 ± 2.5, with a range from min. 0 and max. 9.4 on a scale from 0 to 10 (0: soft; 10: hard). (Supplementary Figure 1). As expected, a significant negative correlation (*p* < 0.0001, *r* = −0.773, *r*^2^ = 0.579) was observed between the variable texture in hand and the water content.

**Figure 4:**
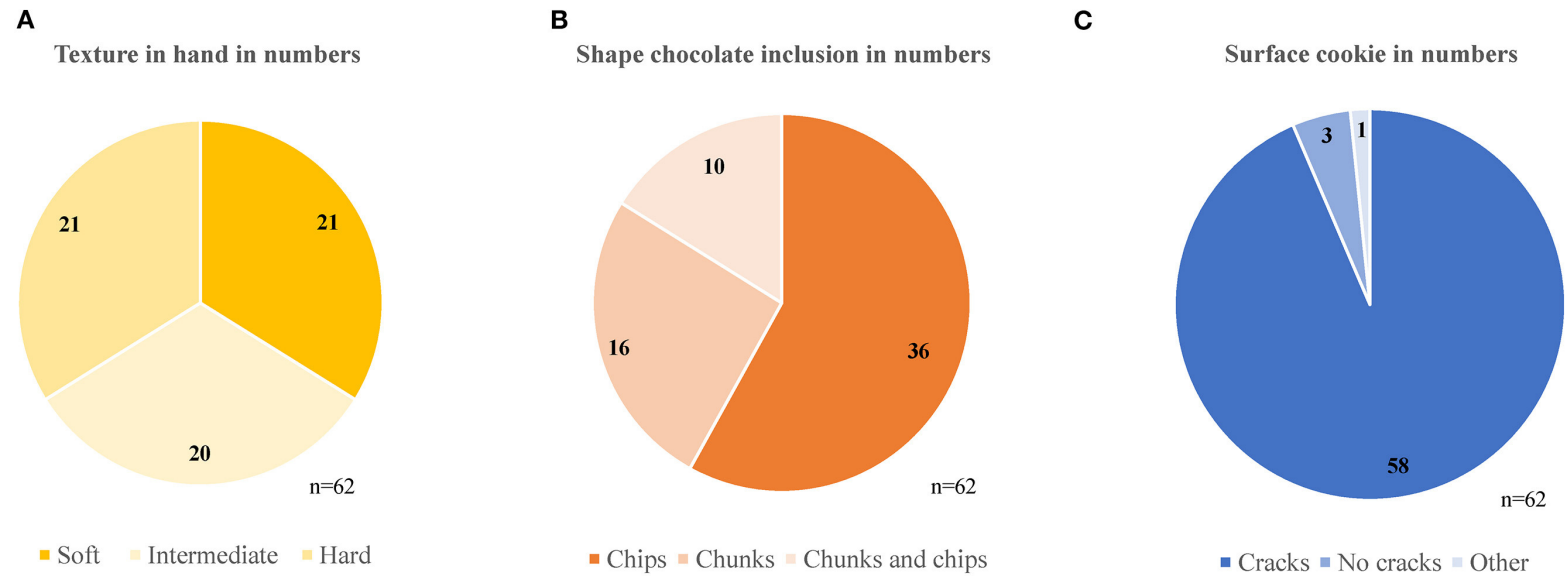
Results of the three sensory screenings on all 62 chocolate chip cookies with the additional ranked criteria **(A)** texture in hand, **(B)** shape of the chocolate inclusion and, **(C)** the surface of the cookie. Those three criteria are part of the 11 additional ranked criteria in order to be able to make an informed subset selection which is representative of the market.

The numbers of cookies for the additional ranking criteria “texture in hand,” “shape chocolate inclusion” and “surface cookie” are shown in Figure 4 with their subgroups. Among the 62 chocolate chip cookies, most products had chips as a chocolate shape and most of the cookies' surfaces were characterized with cracks. All types of cookie textures are well distributed, in soft, intermediate, and hard. All criteria with their subgroups are shown in Supplementary Table 2.

**Figure 5:**
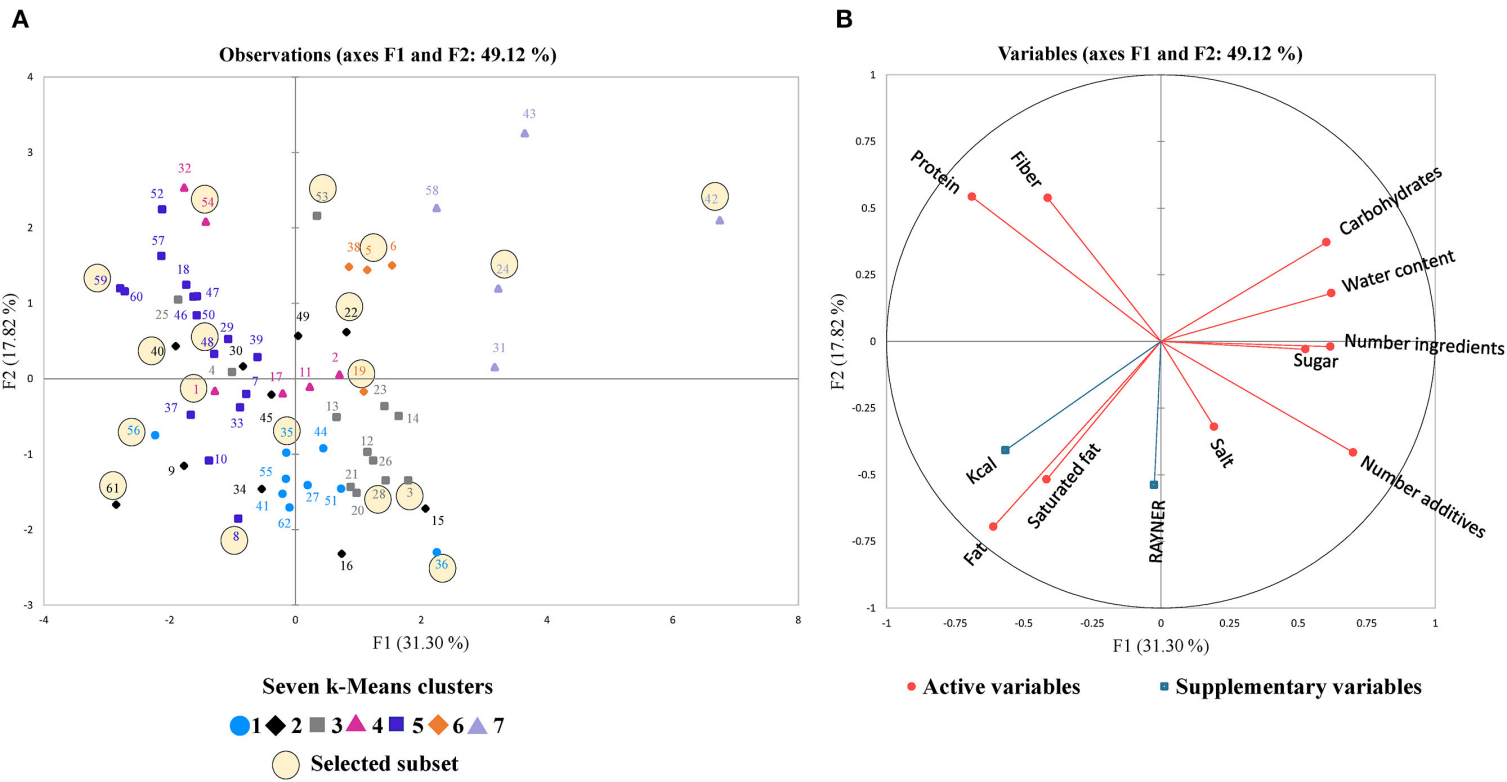
Principal component analysis (PCA) with axes F1 and F2. **(A)** observation plot including 62 cookies with chocolate inclusion colored based on seven different K-means clusters and the 18 selected cookies for the subset in black circles, representative of the market. **(B)** PCA correlation matrix with 10 active nutrition, composition, and water content variables (Fat, saturated fat, carbohydrates, sugar, protein, fiber and salt in g per 100 g, water content in %, number ingredients and number additives), and two supplementary variables (Rayner score, Kcal).

On the PCA score map in Figure 5A, the cookies were grouped and represented with different colors based on seven clusters, obtained by K-means clustering (further variable information in Supplementary Table 1). The broadest variability was explained by axes F1 and F2 with a variance of 49.12%. Results of a conducted ANOVA demonstrated significant differences between almost all clusters (*p* < 0.05), except for the smallest ingredient components fiber and the salt content.

The loadings in Figure 5B show the relationships between 10 active and two supplementary variables. The strongest positive correlations were found between the fat and kcal content (*r* = 0.828), between the Rayner score and the number additives (*r* = 0.538), and between the Rayner score and the salt content (*r* = 0.447). On the other hand, the strongest negative correlations were found between the kcal and water content (*r* = −0.793), the protein content and the number of additives (*r* = −0.659), and between the fat and the water content (*r* = −0.603).

Figure 5A shows broad nutrition, composition, and water content diversity among the 62 chocolate chip cookies. Some cookies on the right side of the plot (axis F1) are characterized by a high carbohydrate, sugar, and water content and a higher number of total additives (technological and sensory) and ingredients. On the left side (axis F1), some cookies are characterized by their high protein and kcal content. Furthermore, cookies on the bottom (axis F2) had higher fat and saturated fat content with a higher Rayner score. Cookies on the top (axis F2) tend to have a higher fiber content. Moreover, and as expected, the Rayner score showed a positive correlation with fat content (*r* = 0.288), saturated fat (*r* = 0.538) and salt content (0.447), and a negative correlation with the fiber content (*r* = −0.281). Cookies of clusters 1 and 3 had a high Rayner score with high fat, saturated fat, and lower fiber content. On the other hand, most of the cookies belonging to clusters 5, 6, and 7 presented a lower Rayner score with higher fiber content, and lower fat and saturated fat content than other cookies. Cookies from cluster 5 showed the highest kcal content, while those of cluster 7 were characterized with high sugar content.

**Figure 6:**
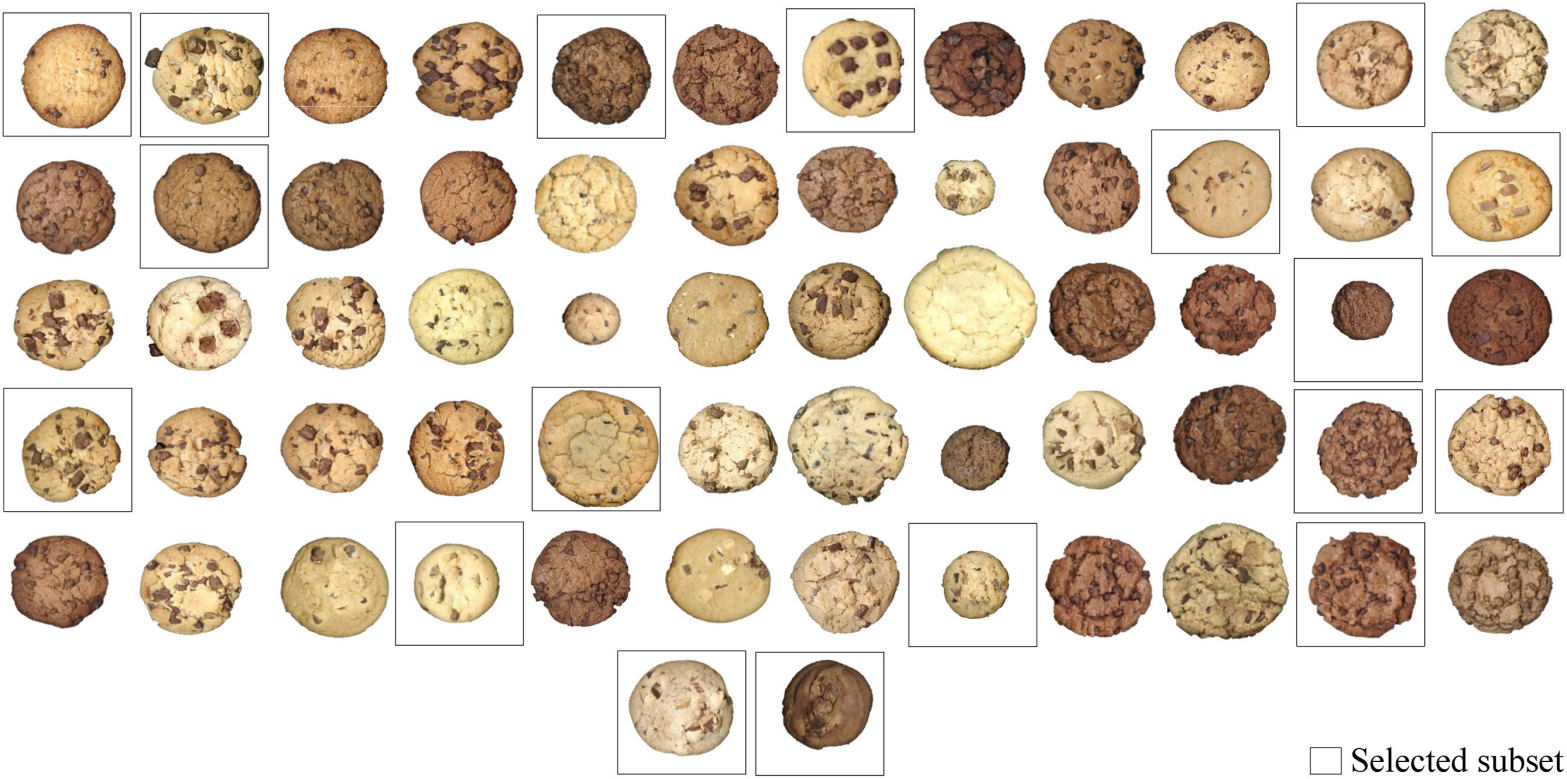
This figure shows 62 pictures of the commercial chocolate chip cookies, including the selected subset of 18 cookies.

Figure 6 presents all 62 commercial chocolate chips cookies. Although this study concentrated on a single product category only, we can observe a broad visual cookie variety. Notably, they differ in their size, their dough color, the shape of the chocolate inclusion, the quantity of the chocolate inclusion appearing on the surface, or the cracks on the cookie surface.

### The Selected Subset and Its Representativity

To best represent the diversity of all chocolate chip cookies, a subset of 18 cookies was also proposed. This accounts for almost a third of the initial 62 cookies. In total, 3 cookies were selected in clusters 1, 2, 3, and 5, while 2 cookies were selected in clusters 4, 6, and 7 (Figure 5 and Supplementary Table 4).

#### Labels and Retailers

**Figure 7:**
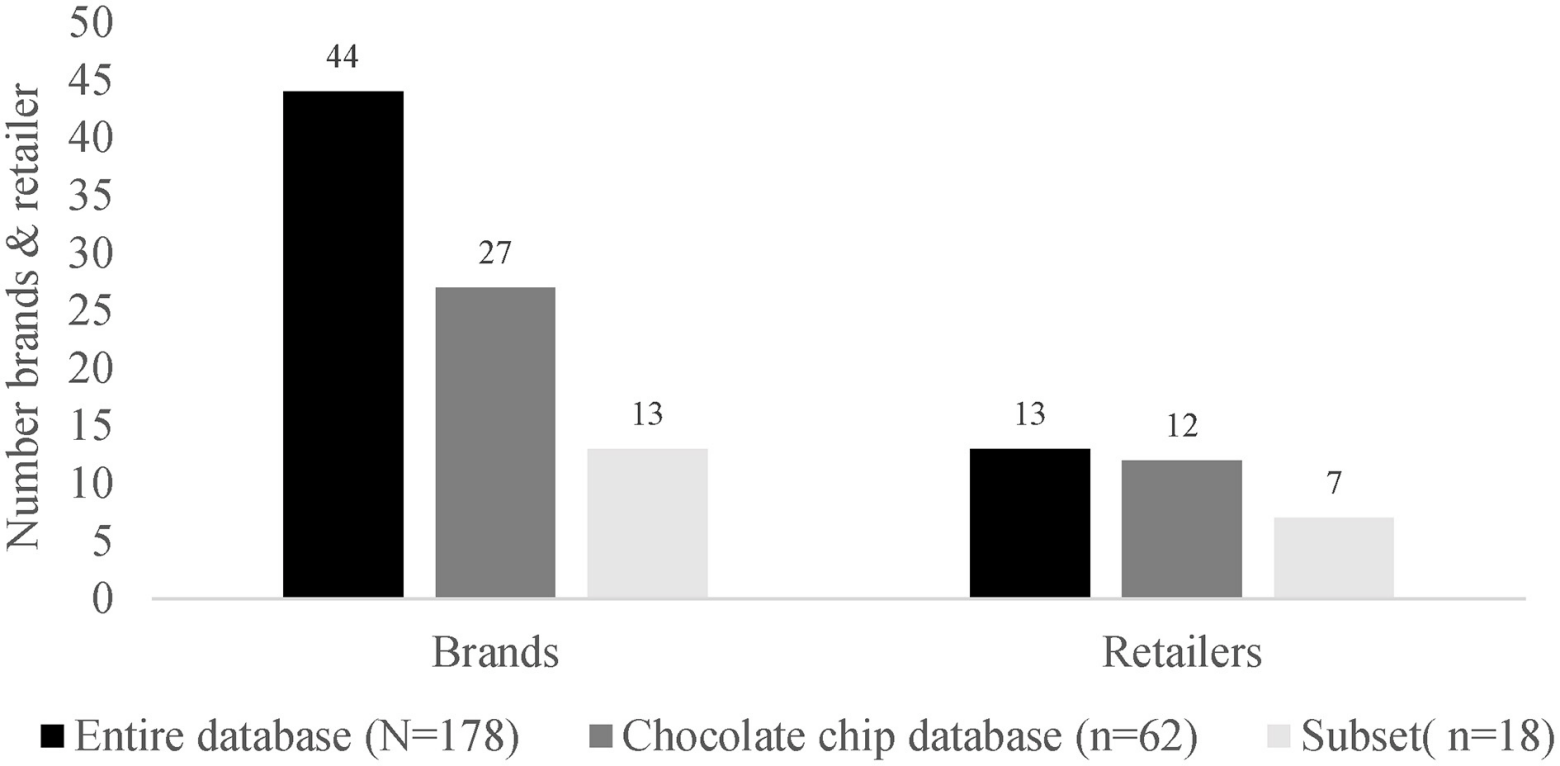
Representativity of the brands and retailers of the selected subset.

The subset of cookies included 13 different private and international labels from 7 retailers. In comparison to the entire cookie database with 178 products, this is almost on third (29.5%) of all labels and more than half (53.8%) of all retailers. Considering the chocolate chip cookie database, the subset included about half (48.1%) of all labels and more than half (58.3%) of all retailers (Figure 7).

#### Quantitative Nutrition, Composition, and Water Content Variables

The 18 selected cookies are highlighted on the PCA of the 62 chocolate chip cookies in Figure 5A. The subset shows broad nutrition, composition, and water content diversity based on 12 plotted variables. Moreover, the selected cookies of each cluster showed a balanced distribution of extreme cookies within and between clusters based on axes F1 and F2.

**Figure 8:**
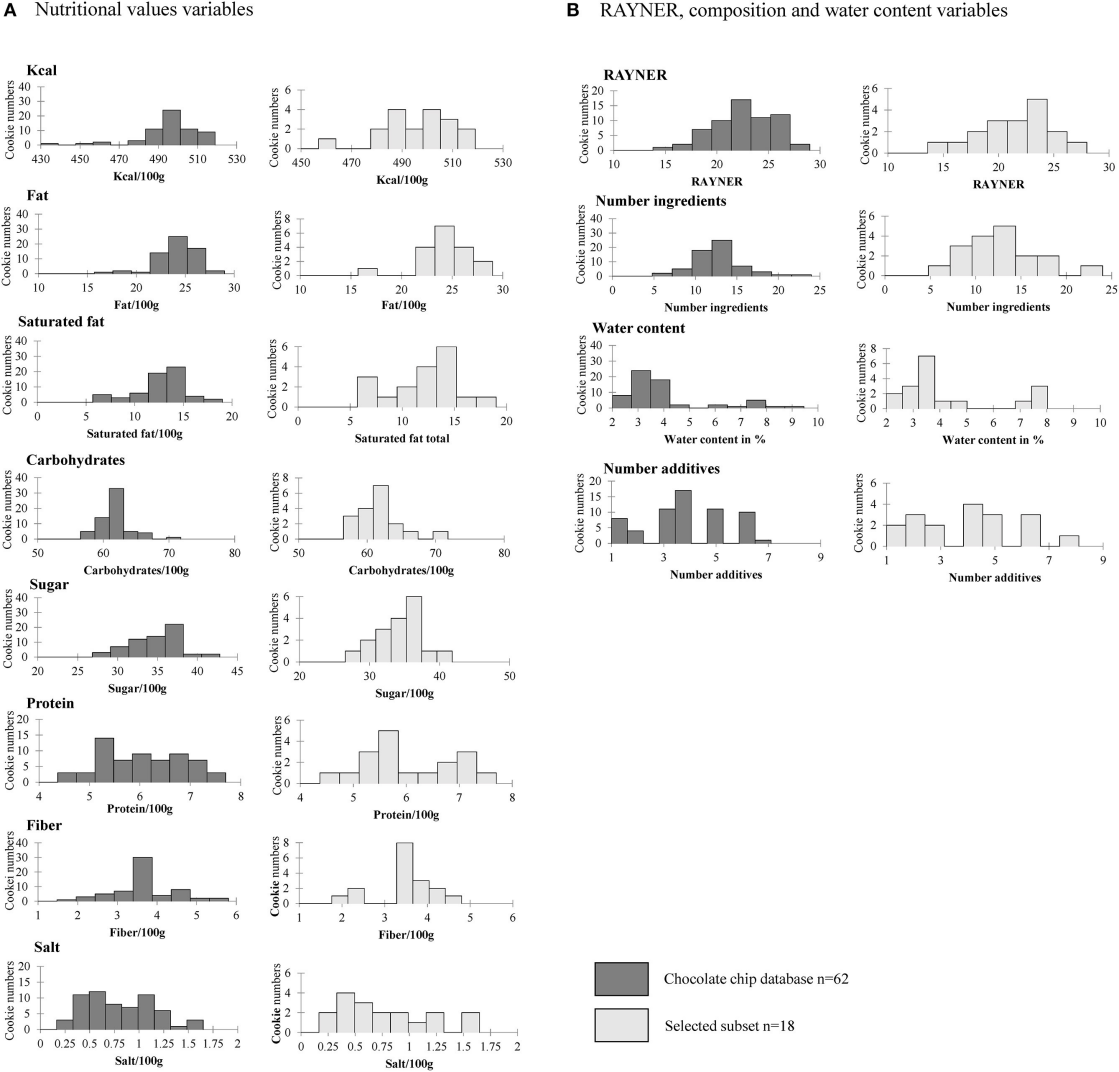
Comparison of 12 nutrition, composition and water content variables among chocolate chip cookies and the selected subset based on distributions with **(A)** nutritional values variables and **(B)** Rayner score, composition, and water content variables.

As can be seen in Figure 8 we observed a similar distribution between the chocolate chips cookies and the selected subset for almost all variables. The subset led to a slightly different distribution for the kcal, fiber, protein, salt, and water content. The subset also accounted for minimal and maximum values for most of the variables.

#### Additional Ranking Criteria

As shown in Supplementary Table 5, the selected subset considered 37 out of the 40 subgroups. Three subgroups (criterion type sugar: syrup, criterion amount chocolate, and cacao powder: middle and criterion weight cookie: high) remained unrepresented. Although we did not seek strict representativeness in terms of group sizes, it can be noted that larger subgroups in the chocolate chip cookie database also had more products in the selected subset. The selected subset contained cookies as well with smaller subgroups in the chocolate chip cookie database, such as the subgroup “type of flour, mixed,” several subgroups from the criteria “amount chocolate and cacao powder” and “surface cookie” and as well the subgroup “type of chocolate, milk.” In addition, each additional ranked criterion was considered when selecting the cookies from the seven clusters.

## Discussion

### Focus on a Single Product Category (Cookie) and Specific Cookie Variety (Chocolate Chip Cookies)

Inventorying one product category only is not so frequent in the field of food reformulation. Further, sensory studies often usually deal with a small number of products without considering the full diversity of the market. However, this study showed that working with a single product category has several advantages.

First of all, focusing on a single product category allows increasing the product number in a database, which will lead to an increased product diversity what is important for the later reformulation. As well conducting analyses implies working on physically available products from the market, as that information is not available on the packaging.

Moreover, it is possible to compare food products' nutritional, sensory, and water content characteristics. Although this study focused on chocolate chip cookies only, it was shown that this product category provided large nutrition, composition, water content, and sensory diversity. Those various characteristics are necessary to better understand the links between products' composition, perception, and liking in a later step. Although focusing on cookies only, the measured water content ranged from 2 to almost 10%, with significant consequences on the perceived texture in hand. Further, it was observed that cookies with an increased kcal content had a lower water content and were perceived as harder in hand. A possible explanation might be due to baking parameters, with a higher water loss during baking leading to an increased kcal content and more hard texture. On the opposite, cookies with a higher water content had either a lower baking time or temperature, which lead to a lower kcal content with a softer texture. Besides the baking parameters, an increased sugar content for clusters 7 might as well lead to a softer texture as sugar is bringing moisture to the cookie dough. Therefore, we suggest that the water content provides important information when it comes to food reformulation among baked cereal products.

A past study showed difficulties in comparing the kcal content and setting cut-offs for nutritional values among different product categories, as the products' composition and the portion sizes were too different (3). Comparing only one product category would make it possible to compare all nutritional values and might help therefore setting pertinent cut-off levels for reformulation.

Focusing on cookies with chocolate chips and excluding the other types of cookies, presents the advantage to have a realistic overview of the range of products, but also led to a slightly decreased range for minimum and maximum nutritional values in this study. The used approach might be more inclusive compared with other studies that usually focus on a specific product or a specific ingredient category (29, 30). Moreover, other studies prioritized leading brands, high price segments, or premium and private label brands for their dataset (31, 32). Considering only market leader brands might have relieved cookies availability in our study. However, we suggest that especially recipes from different brands, including the leader and niche brands, may provide a more diverse product variety and represents the current market for one specific product category.

### The Selected Subset and Its Representativity

This study illustrated how a representative subset of products can be selected from detailed market inventory, including multiple criteria by going beyond the nutrition and composition information on the packaging. The selected subset of 18 cookies in this study is representative of 23 variables and criteria. Including different retailers, private labels and international brands might contribute to a broader range of recipes and product diversity in the subset as when only focusing on selected retailers and international or private labels.

This PCA permits to visually assess that the selected subset was representative of the market, with the presence of “extreme” cookies within a single cluster but as well between the seven clusters. However, only two cookies were selected from cluster 7, although this cluster showed the highest variance. Due to the unavailability of some of the corresponding brands on the market, it was not possible to select more cookies from cluster 7. To avoid this situation, we could have excluded cookies that are difficult to purchase from the start. However, this would have limited our knowledge of the market. In addition to this multifactorial analysis, the unidimensional distributions confirmed the good representativity of the subset for almost all 13 variables.

The results in this study showed that almost all subgroups were represented in the selected subset. Several subgroups remained unselected, although they were higher ranked than the remaining criteria. However, these subgroups had already a low representativity in the chocolate chip cookie database, which increases the difficulty to be selected. One possible approach to increase the chance for a selection even for weakly represented subgroups would be to create subgroups based on extreme values, rather than the calculation of balanced quintiles ranks. Moreover, each criterion was considered for selecting cookies among the clusters. However, the selected cookie numbers were not higher among criteria that were higher ranked. Instead, more cookies were selected on lower-ranked criteria. A possible way to solve this disbalance would be to set higher numbers of cookies to be selected at higher-ranked criteria and lower numbers of cookies to be selected at lower-ranked criteria.

The representativity step is a critical step as the main purpose is primarily to help industrials and decision-makers to anticipate the levers of reformulation. Therefore, working with a reduced subset that is representative of the market is required to conduct further in-depth sensory, physicochemical, and liking analyses.

### Potential for Reformulation Among the Product Category Commercial “Chocolate Chip Cookies” and Prospects for the Future Reformulation Work

We found a large heterogeneity among the chocolate chip cookies in terms of nutritional, compositional, water content, and sensory aspects. Large ranges for minimum and maximum values for fat and sugar indicate a potential for certain commercial cookies to reduce their fat and sugar content. Likewise, we identified the potential to increase the fiber content among certain cookies. Moreover, most of the cookies were graded at the highest Nutri-Score (E), which is associated with poor nutritional quality food. However, this study identified commercial cookies with a reduced Rayner score (but only one cookie with a reduced Nutri-Score D), which is associated with lower fat, saturated fat, and higher fiber content. This rise the question of whether the Rayner respectively the Nutri-Score is an optimal tool to reformulate among a product category which is known to have very high sugar and fat contents, as the effect of the sugar and fat reduction on the score itself might be low. On the other hand, a lump of sugar and fat reduction in too large steps implies many constraints. Therefore, besides nutritional values, further parameters such as the level of processing and the number of additives should be considered for reformulation.

Sweet biscuits are important contributors to children's unhealthy diet, composed of high caloric, highly processed, and palatable foods (6, 33–35). Our data confirm the interest and the possibilities of reformulation among the commercial “chocolate chip cookies” product category. There clearly is scope for improvement of some cookies' nutritional profile, especially by reducing fat and sugar content and increasing fiber content.

Besides the nutritional diversity, we identified three types of cookie textures: soft, intermediate, and hard. Texture properties play a key role in sensory perception and as a driver of preferences for food choices (36, 37). Moreover, besides nutrient manipulation, studies have shown that texture modification might be successful to decrease the “obesogenic” eating style. Indeed, food oral processing, impacted by texture, could influence satiety and satiation of individuals with faster eating rates and shorter oral exposure time (38). This confirms the high interest to expand nutritional and compositional databases with water content variables and sensory criteria.

Eventually, we will use the selected subset to identify future reformulation levers, while maintaining sensory perception and liking. This will imply further in-depth sensory and physicochemical analyses, in order to better understand the link between products composition, sensory and physicochemical properties. This understanding is crucial for a successful reformulation, as this multicriteria approach anticipates potential limitations among the reformulation process.

## Conclusion

The creation of a database based on a comprehensive market inventory with a focus on a single product category and that considers multiple variables allows to describe and compare the diversity of products. It also sets the basis for future reformulation. Besides nutrition and composition information collected from packaging, simple additional characterizations are useful to better assess product diversity. This allowed making an informed and representative subset selection whose representativity was checked by uni- and multi-variate analyses.

Thanks to this subset selection, it will be possible to conduct in-depth sensory, physicochemical, and hedonic investigations that are required to successfully reformulate the products as one possible answer to the improvement of the food offer. Besides nutrient modification, texture modification with possible impact on the oral process, satiation, and satiety is a further promising reformulation lever.

It is also necessary to create a multicriteria database to ideally identify some healthier formulation solutions which minimize changes in sensory perception, liking, and cost-effectiveness. Increasing the potential for voluntary reformulation among industries on one hand and providing a tool to drive public policies, on the other hand, might strengthen the reformulation as an even more impactful lever to enhance our food environment.

## Data Availability Statement

The original contributions presented in the study are included in the article/Supplementary Material, further inquiries can be directed to the corresponding author.

## Author Contributions

CL: conceptualization, data curation, formal analysis, investigation, visualization, and writing–original draft. JD and AS-E: conceptualization, funding acquisition, supervision, and writing–review and editing. VB and IS: conceptualization, investigation, supervision, writing–review, and editing. All authors contributed to the article and approved the submitted version.

## Funding

This project was supported by the international STOP project (Science and Technology in childhood Obesity Policy) which has received funding from the European Union's Horizon 2020 research and innovation program under grant agreement No. 774548.

## Conflict of Interest

The authors declare that the research was conducted in the absence of any commercial or financial relationships that could be construed as a potential conflict of interest.

## Publisher's Note

All claims expressed in this article are solely those of the authors and do not necessarily represent those of their affiliated organizations, or those of the publisher, the editors and the reviewers. Any product that may be evaluated in this article, or claim that may be made by its manufacturer, is not guaranteed or endorsed by the publisher.
