# Supplementary material for: “How to Select a Representative Product Set From Market Inventory?” A Multicriteria Approach as a Base for Future Reformulation of Cookies
Source: Front Nutr. 2022 Jan 24;8:749596. doi: 10.3389/fnut.2021.749596 (PMC8819632; doi:10.3389/fnut.2021.749596)
Supplement: Supplementary file 1 [file Data_Sheet_1.docx]

Supplementary Tables:

Supplementary Table 1: Nutrition (per 100g), composition and water content information among the chocolate chip cookie database. Eleven quantitative variables (fat, sfa, carbohydrates, sugar, protein, fiber, salt, ingredients, technological additives, sensory additives, water content) were included for the clustering, while 12 quantitative variables (kcal, fat, sfa, carbohydrates, sugar, protein, fiber, salt, Rayner score, ingredients, additives, water content) were included for the representativity check. With * for the selected cookie subset and with ** for the saturated fat content per 100g.

| Cookie | Kcal | | Fat  g | Sfa  **  g | Carbo-hydrates  g | Sugar  g | Protein  g | Fiber  g | Salt  g | Rayner score | Ingre-dients | Techno-logical additives | Sensory additives | Addi-tives | Water content  % |
| --- | --- | --- | --- | --- | --- | --- | --- | --- | --- | --- | --- | --- | --- | --- | --- |
| 1* | 503 | 25 | | 12 | 62 | 27 | 6.5 | - | 1.55 | 27 | 11 | 3 | 0 | 3 | 3.63 |
| 2 | 495 | 23 | | 11.5 | 65.6 | 31.1 | 6.5 | 2.6 | 1.5 | 26 | 12 | 4 | 0 | 4 | 3.33 |
| 3* | 509 | 24.9 | | 13.3 | 64.3 | 38.3 | 5.2 | 3.6 | 1.5 | 25 | 12 | 5 | 1 | 6 | 3.87 |
| 4 | 508 | 26 | | 11 | 61 | 34 | 6.4 | 4.6 | 1.4 | 24 | 12 | 3 | 0 | 3 | 3.04 |
| 5* | 504 | 23 | | 6.6 | 66 | 37 | 6.1 | 4.4 | 1.3 | 20 | 9 | 5 | 0 | 5 | 2.18 |
| 6 | 504 | 23 | | 6.6 | 66 | 37 | 6.1 | 4.4 | 1.3 | 20 | 11 | 5 | 0 | 5 | 3.1 |
| 7 | 497 | 24.4 | | 13.2 | 61 | 32.7 | 6.5 | 3.6 | 1.24 | 23 | 13 | 3 | 0 | 3 | 2.94 |
| 8* | 495 | 26 | | 13 | 58 | 32 | 5.5 | 3.5 | 1.2 | 24 | 10 | 5 | 0 | 5 | 2.65 |
| 9 | 504 | 26 | | 15 | 59 | 34 | 6.5 | - | 1.18 | 24 | 6 | 4 | 0 | 4 | 3.29 |
| 10 | 498 | 26 | | 13 | 58 | 32 | 6 | 4 | 1.17 | 23 | 11 | 4 | 0 | 4 | 3.08 |
| 11 | 499 | 23.9 | | 12.2 | 64.9 | 28.3 | 5.3 | - | 1.15 | 27 | 12 | 4 | 0 | 4 | 3.53 |
| 12 | 490 | 24.6 | | 11.6 | 61.8 | 37.2 | 5.4 | - | 1.1 | 28 | 13 | 5 | 1 | 6 | 2.81 |
| 13 | 499 | 24.6 | | 11.6 | 61.8 | 37.2 | 5.4 | 4.5 | 1.1 | 23 | 11 | 5 | 1 | 6 | 2.69 |
| 14 | 494 | 24 | | 8.9 | 62 | 37 | 5.2 | 3.2 | 1.1 | 22 | 13 | 4 | 1 | 5 | 3.54 |
| 15 | 484 | 24 | | 15 | 62 | 34 | 4.8 | 2.7 | 1.1 | 24 | 12 | 6 | 0 | 6 | 7.38 |
| 16 | 504 | 26 | | 14 | 61 | 36 | 4.5 | 2.9 | 1.05 | 23 | 10 | 5 | 0 | 5 | 3.68 |
| 17 | 504 | 24 | | 13 | 64 | 31 | 5.8 | 3.5 | 1.04 | 22 | 11 | 4 | 0 | 4 | 2.56 |
| 18 | 498 | 23.2 | | 14.7 | 62.2 | 33.3 | 7.6 | - | 1.03 | 27 | 10 | 1 | 0 | 1 | 3.68 |
| 19* | 497 | 24 | | 9.6 | 63 | 37 | 5.3 | 3.9 | 1 | 22 | 8 | 5 | 1 | 6 | 3.17 |
| 20 | 500 | 25 | | 13 | 62 | 36 | 5.3 | 3.1 | 1 | 23 | 12 | 5 | 1 | 6 | 3 |
| 21 | 501 | 25 | | 13 | 62 | 35 | 5.3 | 3.2 | 1 | 23 | 12 | 5 | 1 | 6 | 3.25 |
| 22* | 479 | 23 | | 14 | 61 | 35 | 5.6 | 3.7 | 0.18 | 17 | 11 | 3 | 1 | 4 | 7.88 |
| 23 | 498 | 24.2 | | 10.1 | 63.4 | 36.6 | 5.3 | 3.3 | 0.92 | 23 | 13 | 4 | 1 | 5 | 3.09 |
| 24* | 478 | 22 | | 7.4 | 63 | 37 | 5.8 | - | 0.9 | 22 | 16 | 6 | 0 | 6 | 7.85 |
| 25 | 501 | 25 | | 12 | 59 | 35 | 7.3 | 5.5 | 0.88 | 21 | 12 | 4 | 0 | 4 | 2.41 |
| 26 | 501 | 25 | | 12 | 62 | 37 | 5.4 | 3.3 | 0.87 | 23 | 13 | 5 | 1 | 6 | 3.53 |
| 27 | 503 | 26 | | 15 | 60 | 34 | 5.4 | - | 0.84 | 26 | 19 | 4 | 0 | 4 | 3.73 |
| 28* | 485 | 24 | | 12 | 61 | 36 | 5.2 | 1.8 | 0.83 | 24 | 13 | 4 | 0 | 4 | 4.56 |
| 29 | 516 | 24.4 | | 13.6 | 62.7 | 31.8 | 7.4 | 3.6 | 0.82 | 21 | 12 | 4 | 0 | 4 | 3.15 |
| 30 | 511 | 25 | | 15.9 | 63 | 38.7 | 6.9 | - | 0.8 | 27 | 13 | 2 | 0 | 2 | 2.55 |
| 31 | 452 | 21 | | 12 | 60 | 36 | 5.1 | 3.3 | 0.71 | 21 | 16 | 6 | 0 | 6 | 9.36 |
| 32 | 492 | 23 | | 8.7 | 61.2 | 27.2 | 7.2 | 5.2 | 0.7 | 18 | 12 | 3 | 0 | 3 | 3.46 |
| 33 | 494 | 24.1 | | 16.6 | 61.4 | 32.6 | 6.7 | - | 0.7 | 26 | 11 | 5 | 0 | 5 | 3.46 |
| 34 | 511 | 26.9 | | 13.7 | 61.1 | 34.1 | 4.5 | - | 0.7 | 26 | 10 | 3 | 0 | 3 | 3.3 |
| 35* | 500 | 26 | | 14 | 59 | 35 | 5.6 | 4 | 0.66 | 20 | 18 | 3 | 1 | 4 | 3.53 |
| 36* | 485 | 25 | | 14 | 60 | 33 | 4.7 | 2.4 | 0.75 | 23 | 17 | 6 | 1 | 7 | 6.83 |
| 37 | 506 | 26.3 | | 17.4 | 62.5 | 31.5 | 6.8 | - | 0.65 | 25 | 13 | 3 | 0 | 3 | 3.47 |
| 38 | 493 | 23 | | 6.6 | 63 | 37 | 6.5 | 3.8 | 0.65 | 17 | 11 | 5 | 0 | 5 | 2.88 |
| 39 | 488 | 23.1 | | 14.8 | 61.9 | 32.4 | 6.4 | - | 0.65 | 25 | 11 | 4 | 0 | 4 | 3.54 |
| 40* | 511 | 25.1 | | 16.1 | 62.2 | 36.9 | 7 | - | 0.64 | 26 | 9 | 1 | 0 | 1 | 2.74 |
| 41 | 496 | 26 | | 15 | 58 | 37 | 5.2 | - | 0.64 | 26 | 13 | 4 | 0 | 4 | 4.22 |
| 42* | 458 | 17.1 | | 9.5 | 70.8 | 40.8 | 4.8 | 2.2 | 0.6 | 22 | 23 | 5 | 0 | 5 | 7.93 |
| 43 | 465 | 19.2 | | 10.6 | 67.3 | 41.8 | 5.3 | 5.7 | 0.5 | 21 | 15 | 5 | 0 | 5 | 7.56 |
| 44 | 498 | 26 | | 14 | 59 | 38 | 6.2 | 3.4 | 0.59 | 22 | 20 | 3 | 1 | 4 | 4.29 |
| 45 | 493 | 25 | | 16 | 61 | 37 | 6.1 | - | 0.56 | 26 | 10 | 3 | 0 | 3 | 5.79 |
| 46 | 486 | 23 | | 14 | 61 | 31 | 6.6 | - | 0.55 | 24 | 12 | 1 | 0 | 1 | 3.6 |
| 47 | 486 | 23 | | 14 | 61 | 31 | 6.6 | - | 0.53 | 24 | 12 | 1 | 0 | 1 | 3.36 |
| 48* | 518 | 26 | | 14 | 63 | 33 | 6.9 | 4.1 | 0.52 | 20 | 11 | 4 | 0 | 4 | 3.26 |
| 49 | 481 | 23 | | 15 | 61 | 37 | 6.1 | - | 0.48 | 26 | 9 | 3 | 0 | 3 | 6.34 |
| 50 | 518 | 25.7 | | 13.4 | 62.1 | 33.9 | 7.2 | 4.6 | 0.46 | 20 | 11 | 4 | 0 | 4 | 3.08 |
| 51 | 509 | 26.2 | | 14.2 | 61.3 | 37.7 | 5.9 | 2.6 | 0.46 | 23 | 16 | 4 | 1 | 5 | 3.57 |
| 52 | 485 | 23 | | 12 | 60 | 34 | 7 | 5 | 0.44 | 19 | 10 | 1 | 0 | 1 | 3.18 |
| 53* | 488 | 22 | | 9 | 64 | 36 | 6.7 | 3.4 | 0.44 | 18 | 13 | 2 | 0 | 2 | 3.32 |
| 54* | 489 | 24 | | 5.9 | 59 | 30 | 7.6 | 3.5 | 0.43 | 14 | 14 | 2 | 0 | 2 | 3.65 |
| 55 | 511 | 26.2 | | 14.6 | 61.6 | 32.8 | 6 | 2.4 | 0.43 | 21 | 16 | 3 | 1 | 4 | 3.25 |
| 56* | 514 | 28 | | 13 | 57 | 34 | 7 | - | 0.42 | 24 | 15 | 3 | 0 | 3 | 2.47 |
| 57 | 493 | 24 | | 13 | 60 | 33 | 6.9 | 4.6 | 0.41 | 19 | 11 | 1 | 0 | 1 | 3.65 |
| 58 | 433 | 18 | | 10.3 | 59.9 | 37.4 | 6 | 3.1 | 0.4 | 19 | 13 | 3 | 0 | 3 | 8.33 |
| 59* | 501 | 25.2 | | 14.1 | 59.3 | 30.7 | 7 | 4.7 | 0.39 | 18 | 12 | 1 | 0 | 1 | 3.67 |
| 60 | 496 | 25 | | 13 | 59 | 31 | 6.6 | 4.5 | 0.3 | 18 | 9 | 1 | 0 | 1 | 3.78 |
| 61* | 511 | 28 | | 18 | 57 | 35 | 5.8 | - | 0.3 | 24 | 7 | 2 | 0 | 2 | 3.49 |
| 62 | 497 | 26.2 | | 14.6 | 59.6 | 35.9 | 5.7 | 2.6 | 0.89 | 23 | 15 | 3 | 0 | 3 | 3.68 |

Supplementary Table 2: Eleven ranked criteria (1-11) with the 40 subgroups and the constraint “availability” among the 62 chocolate chip cookies.

|  |  |  |  |  |  |  |  |  |  |  |  |  |  |  |
| --- | --- | --- | --- | --- | --- | --- | --- | --- | --- | --- | --- | --- | --- | --- |
|  | **11 Criteria with subgroups**** | | | | | | | | | | | | |  |
|  | **Cookie** | **Con-staint** | **1** | **2** | **3** | **4** | **5** | **6** | **7** | **8** | **9** | **10** | **11** |  |
|  | 1* | ok | wheat | crystal | veg. | - | very l. | dark | soft | chips | cracks | low | low |  |
|  | 2 | ok | wheat | crystal | veg. | - | very l. | dark | hard | chips | cracks | low | very l. |  |
|  | 3* | ok | wheat | crystal | veg. | - | very h. | dark | intermed. | chunks | cracks | mid. | low |  |
|  | 4 | not ok | wheat | crystal | mix. | - | very h. | dark | hard | chips & chunks | cracks | mid. | high |  |
|  | 5* | ok | wheat | crystal | veg. | very h. | mid. | dark | hard | chips | cracks | low | very l. |  |
|  | 6 | ok | wheat | crystal | veg. | very h. | mid. | dark | hard | chips | cracks | mid. | very l. |  |
|  | 7 | ok | wheat | crystal | veg. | low | mid. | dark | intermed. | chips | cracks | low | very l. |  |
|  | 8* | ok | wheat | crystal | veg. | high | low | dark | soft | chips | cracks | low | very l. |  |
|  | 9 | ok | wheat | crystal | veg. | - | low | dark | intermed. | chips | cracks | low | very l. |  |
|  | 10 | ok | wheat | crystal | veg. | high | low | dark | intermed. | chips | cracks | low | low |  |
|  | 11 | not ok | wheat | crystal | veg. | - | very l. | dark | soft | chips | cracks | low | very l. |  |
|  | 12 | not ok | wheat | crystal | mix. | - | very h. | dark | intermed. | chunks | cracks | mid. | low |  |
|  | 13 | not ok | wheat | crystal | mix. | - | low | dark | intermed. | chips & chunks | cracks | mid. | mid. |  |
|  | 14 | ok | wheat | crystal | mix. | - | very h. | dark | hard | chips & chunks | cracks | mid. | low |  |
|  | 15 | ok | wheat | mixed | ani. | - | very l. | dark | soft | chunks | cracks | very h. | high |  |
|  | 16 | ok | wheat | crystal | veg. | - | high | dark | intermed. | chunks | cracks | low | mid. |  |
|  | 17 | not ok | wheat | mixed | veg. | - | - | dark | hard | chips | cracks | very l. | high |  |
|  | 18 | not ok | mixed | crystal | ani. | - | low | dark | intermed. | chips | no cracks | very l. | very h. |  |
|  | 19* | ok | wheat | crystal | mix. | - | very h. | dark | soft | chips & chunks | cracks | mid. | very l. |  |
|  | 20 | ok | wheat | crystal | veg. | - | very h. | dark | intermed. | chips & chunks | cracks | mid. | low |  |
|  | 21 | ok | wheat | crystal | veg. | - | high | dark | intermed. | chips & chunks | cracks | mid. | low |  |
|  | 22* | ok | wheat | mixed | ani. | low | very l. | dark | soft | chunks | other | very h. | high |  |
|  | 23 | ok | wheat | crystal | mix. | - | very h. | dark | hard | chips & chunks | cracks | mid. | low |  |
|  | 24* | ok | wheat | crystal | veg. | - | very l. | dark | soft | chunks | cracks | very h. | very h. |  |
|  | 25 | not ok | wheat | crystal | veg. | low | mid. | dark | intermed. | chips | cracks | very l. | very l. |  |
|  | 26 | ok | wheat | crystal | mix. | - | high | dark | soft | chips & chunks | cracks | mid. | mid. |  |
|  | 27 | not ok | wheat | crystal | mix. | - | low | dark | hard | chunks | cracks | very h. | high |  |
|  | 28* | ok | wheat | mixed | veg. | - | high | milk | soft | chips | cracks | very l. | mid. |  |
|  | 29 | ok | wheat | crystal | veg. | low | low | dark | intermed. | chips | cracks | low | very l. |  |
|  | 30 | not ok | wheat | crystal | ani. | - | low | mix. | intermed. | chips | no cracks | very l. | very h. |  |
|  | 31 | not ok | wheat | mixed | mix. | very h. | - | dark | soft | chunks | cracks | very h. | high |  |
|  | 32 | not ok | wheat | mixed | veg. | mid. | low | dark | hard | chips | cracks | mid. | very h. |  |
|  | 33 | ok | wheat | crystal | ani. | - | mid. | dark | intermed. | chips | cracks | mid. | very h. |  |
|  | 34 | not ok | wheat | syrop | veg. | - | - | dark | hard | chips | cracks | mid. | very l. |  |
|  | 35* | ok | wheat | mixed | mix. | - | very h. | mix. | soft | chips & chunks | cracks | mid. | low |  |
|  | 36* | ok | wheat | mixed | mix. | - | very l. | dark | soft | chunks | cracks | very h. | high |  |
|  | 37 | not ok | wheat | crystal | ani. | - | low | mix. | soft | chips | cracks | very l. | very h. |  |
|  | 38 | not ok | wheat | crystal | veg. | - | high | dark | hard | chips | cracks | very l. | very l. |  |
|  | 39 | ok | wheat | crystal | ani. | very l. | low | dark | intermed. | chips | cracks | mid. | very h. |  |
|  | 40* | ok | wheat | crystal | ani. | - | low | dark | intermed. | chips | no cracks | very l. | very h. |  |
|  | 41 | ok | wheat | crystal | mix. | - | very h. | dark | soft | chips | cracks | very h. | high |  |
|  | 42* | ok | wheat | mixed | mix. | - | very l. | milk | soft | chunks | cracks | very h. | high |  |
|  | 43 | not ok | wheat | crystal | mix. | - | very l. | dark | soft | chunks | cracks | very h. | high |  |
|  | 44 | not ok | mixed | mixed | mix. | - | very h. | dark | hard | chips | cracks | high | low |  |
|  | 45 | ok | wheat | crystal | ani. | - | low | dark | soft | chunks | cracks | very h. | very h. |  |
|  | 46 | ok | wheat | crystal | ani. | very l. | mid. | dark | hard | chips | cracks | very l. | mid. |  |
|  | 47 | ok | wheat | crystal | ani. | very l. | mid. | dark | hard | chips | cracks | low | mid. |  |
|  | 48* | not ok | wheat | crystal | veg. | high | high | dark | hard | chips | cracks | very l. | mid. |  |
|  | 49 | ok | wheat | crystal | ani. | - | low | dark | soft | chunks | cracks | very h. | very h. |  |
|  | 50 | ok | wheat | crystal | veg. | very h. | mid. | dark | hard | chips | cracks | very l. | mid. |  |
|  | 51 | ok | wheat | crystal | mix. | - | high | mix. | intermed. | chunks | cracks | high | mid. |  |
|  | 52 | ok | wheat | crystal | mix. | very l. | high | dark | hard | chips | cracks | mid. | mid. |  |
|  | 53* | ok | wheat | crystal | veg. | - | high | dark | hard | chips | cracks | very l. | high |  |
|  | 54* | ok | mixed | crystal | veg. | - | very l. | dark | intermed. | chips | cracks | very l. | high |  |
|  | 55 | ok | wheat | crystal | mix. | - | high | mix. | intermed. | chunks | cracks | high | mid. |  |
|  | 56* | ok | mixed | crystal | ani. | - | high | milk | hard | chips | cracks | very l. | mid. |  |
|  | 57 | ok | wheat | crystal | veg. | very l. | mid. | dark | hard | chips | cracks | very l. | low |  |
|  | 58 | not ok | wheat | crystal | mix. | - | very l. | dark | soft | chips & chunks | cracks | very h. | very h. |  |
|  | 59* | not ok | wheat | crystal | veg. | very l. | mid. | dark | intermed. | chips | cracks | high | low |  |
|  | 60 | ok | wheat | crystal | veg. | - | very l. | dark | soft | chips | cracks | low | very l. |  |
|  | 61* | ok | wheat | crystal | ani. | - | high | dark | soft | chunks | cracks | mid. | very h. |  |
|  | 62 | ok | wheat | mixed | mix. | - | very h. | mix. | hard | chips | cracks | high | very h. |  |
|  |  |  |  |  |  |  |  |  |  |  |  |  |  |  |

veg = vegetale, ani = animal, mix. = mixed, very l. = very low, very h. = very high, mid. = middle, intermed. = intermediate

* 18 selected cookies for the subset

**Criteria 1-11: 1 Type of flour, 2 Type of sugar, 3 Type of fat, 4 Cacao & chocolate powder %, 5 Chocolate inclusion %, 6 Type chocolate inclusion, 7 Texture in hand, 8 Shape chocolate inclusion, 9 Surface, 10 Weight cookie g/unit, 11 Price per kg

**Supplementary Table 3:** All cookies which were not available in short time were excluded from the selection. The first criteria was then the type of flour. As it is visible in Supplementary Table 1, the subgroup “mixed” was the only and unique subgroup within this criteria and cluster 1. Therefore, this cookie 56 was selected. Trying to maintain the cookie diversity, all cookies with the same type of sugar as the previous selected were then excluded (crystal sugar), until a subgroups was unique within a criteria (1-11) and until all number of cookies per cluster (1-7) were selected.

| **Cluster 1** | **11 ranked criteria** | | | | | | | | | | | |
| --- | --- | --- | --- | --- | --- | --- | --- | --- | --- | --- | --- | --- |
|  | **avai-lability** | **1. type of flour** | **2. type of**  **sugar** | **3. type of fat** | **4. % powder** | **5. % chocolate** | **6. type of chocolate** | **7. sensory** | **8. shape chocolate chips** | **9. surface** | **10. g/unit** | **11. price** |
| ~~27~~ | ~~not available~~ | ~~wheat~~ | ~~crystal sugar~~ | ~~mixed~~ | ~~-~~ | ~~low~~ | ~~dark~~ | ~~hard~~ | ~~chunks~~ | ~~cracks~~ | ~~very high~~ | ~~high~~ |
| 35 | ok | wheat | mixed with syrop | mixed | - | very high | mixed | soft | chips and chunks | cracks | middle | low |
| 36 | ok | wheat | mixed with syrop | mixed | - | very low | dark | soft | chunks | cracks | very high | high |
| 41 | ok | wheat | crystal sugar | mixed | - | very high | dark | soft | chips | cracks | very high | high |
| ~~44~~ | ~~not available~~ | ~~mixed~~ | ~~mixed with syrop~~ | ~~mixed~~ | ~~-~~ | ~~very high~~ | ~~dark~~ | ~~hard~~ | ~~chips~~ | ~~cracks~~ | ~~high~~ | ~~low~~ |
| 51 | ok | wheat | crystal sugar | mixed | - | high | mixed | Inter-mediate | chunks | cracks | high | middle |
| 55 | ok | wheat | crystal sugar | mixed | - | high | mixed | Inter-mediate | chunks | cracks | high | middle |
| **56** | ok | **mixed** | crystal sugar | animal | - | high | milk | hard | chips | cracks | very low | middle |
| 62 | ok | wheat | mixed with syrop | mixed | - | very high | mixed | hard | chips | cracks | high | very high |

**Supplementary Table 4:** k-Means clustering with seven clusters among 62 cookies. Clusters with highest variances included the lowest cookie numbers and those cookies which were hardly available.

| **Results by k-Means clusters 1-7:** |  |  |  |  |  |  |  |
| --- | --- | --- | --- | --- | --- | --- | --- |
|  | **1** | **2** | **3** | **4** | **5** | **6** | **7** |
|  | 44 | 22* |  | 17 | 48* | 19* | 58 |
|  | 55 | 40* | 12 | 54* | 50 | 38 | 43 |
|  | 51 | 30 | 26 | 1* | 18 | 6 | 31 |
|  | 35* | 9 | 23 | 2 | 57 | 5* | 42* |
|  | 36* | 45 | 14 | 11 | 52 |  | 24* |
|  | 56* | 49 | 53* | 32 | 29 |  |  |
|  | 62 | 34 | 28* |  | 59* |  |  |
|  | 27 | 61* | 25 |  | 37 |  |  |
|  | 41 | 15 | 4 |  | 33 |  |  |
|  |  | 16 | 20 |  | 60 |  |  |
|  |  |  | 21 |  | 46 |  |  |
|  |  |  | 3* |  | 47 |  |  |
|  |  |  |  |  | 7 |  |  |
|  |  |  |  |  | 39 |  |  |
|  |  |  |  |  | 10 |  |  |
|  |  |  |  |  | 8* |  |  |
| Total cookies per cluster | 9 | 10 | 12 | 6 | 16 | 4 | 5 |
| Within cluster variance | 15.428 | 21.577 | 9.925 | 21.260 | 11.698 | 17.406 | 54.409 |
| Total selected cookies per cluster | 3 | 3 | 3 | 2 | 3 | 2 | 2 |

* 18 selected cookies for the subset. Therefore, we selected three cookies per clusters containing higher cookie numbers and two cookies per clusters containing lower cookie numbers

**Supplementary Table 5:** Representativeness of the selected subset based on 11 ranked criteria with subgroups.

| **Criterion/constraint** | **Total cookie numbers** | **Subgroups** | **Cookie numbers in subgroups and % from total cookie numbers** | **Selected cookies in numbers and % from cookie numbers in subgroups** | **Selected cookies per criterion** |
| --- | --- | --- | --- | --- | --- |
| Constraint: availability | 62 | available  not available | 43 (69.4%)  19 (30.6%) | 18 (41.9%)  - | 18 |
| Type flour | 62 | wheat  mixed | 58 (93.5%)  4 (6.5%) | 16 (27.6%)  2 (50%) | 2 |
| Type sugar | 62 | crystal sugar  mixed  syrup | 50 (80.6%)  11 (17.7%)  1 (1.6%) | 13 (26%)  5 (45.5%)  - | 1 |
| Type fat | 62 | vegetale  mixed  animal | 28 (45.2%)  20 (32.3%)  14 (22.6%) | 9 (32.1%)  4 (20%)  5 (35.7%) | 2 |
| Amount chocolate & cacao powder | 18 | very low  low  middle  high  very high | 6 (33.3%)  4 (22.2%)  1 (5.6%)  3 (16.7%)  4 (22.2%) | 1 (16.6%)  1 (25%)  -  2 (66.6%)  1 (25%) | 1 |
| Amount chocolate inclusion | 59 | very low  low  middle  high  very high | 12 (20.3%)  14 (23.7%)  10 (16.9%)  12 (20.3%)  11 (18.6%) | 6 (50%)  2 (14.3%)  2 (20%)  5 (41.6)  3 (27.3%) | 2 |
| Type chocolate | 62 | dark  mixed  milk | 53 (85.5%)  6 (9.7%)  3 (4.8%) | 14 (26.4%)  1 (16.6%)  3 (100%) | 1 |
| Texture cookie | 62 | hard  intermediate  soft | 21 (33.9%)  20 (32.3%)  21 (33.9%) | 4 (19.1%)  4 (20%)  10 (47.6%) | 1 |
| Shape chocolate inclusion | 62 | chips  chips & chunks  chunks | 36 (58.1%)  10 (16.1%)  16 (25.8%) | 10 (27.7%)  2 (20%)  6 (37.5%) | 2 |
| Surface cookie | 62 | cracks  no cracks  other | 58 (93.5%)  3 (4.8%)  1 (1.7%) | 16 (27.6%)  1 (33.3%)  1 (100%) | 1 |
| Weight cookie | 62 | very low  low  middle  high  very high | 15 (24.2%)  12 (19.4%)  18 (29%)  5 (8.1%)  12 (19.4%) | 6 (40%)  4 (33.3%)  4 (22.2%)  -  4 (33.3%) | 3 |
| Price cookie | 62 | very low  low  middle  high  very high | 13 (21%)  12 (19.4%)  12 (19.4%)  12 (19.4%)  13 (21%) | 3 (23.1%)  3 (25%)  3 (25%)  6 (50%)  3 (23.1%) | 2 |
